# P26 Culture-negative infective endocarditis with complete heart block and embolic stroke: diagnostic challenges and antimicrobial stewardship lessons

**DOI:** 10.1093/jacamr/dlaf230.033

**Published:** 2025-12-04

**Authors:** Karim Samak, Rasha Abdelsalam Elshenawy

**Affiliations:** Royal Free Hospital, London, UK; School of Health, Medicine and Life Sciences, University of Hertfordshire, Hatfield, UK

## Abstract

**Background:**

Infective endocarditis (IE) is a serious infection of the heart lining and valves, associated with high morbidity and mortality.^1^ Diagnosis is guided by the Modified Duke’s Criteria, which integrate microbiological, imaging and clinical features, and were most recently updated in 2023.^2^ Culture-negative IE (CNIE) occurs in up to 20% of cases, often after prior antibiotic exposure or infection with fastidious organisms, making diagnosis particularly difficult.^3^ Antimicrobial stewardship (AMS), defined as optimizing antimicrobial use to improve outcomes while minimizing toxicity and resistance, is vital to addressing antimicrobial resistance (AMR).^4^

**Objectives:**

This abstract presents a complex CNIE case with complete heart block (CHB) and embolic stroke, illustrating the role of Duke’s Criteria, multidisciplinary decision-making and AMS.

**Methods:**

A retrospective case review was conducted on a patient admitted in June 2025 with suspected infective endocarditis at an NHS Foundation Trust. Data included clinical presentation, microbiology, imaging, echocardiography, management and multidisciplinary team (MDT) discussions. The case was assessed using the Modified Duke’s Criteria to establish diagnostic certainty and guide clinical decision-making.

**Results:**

An 85-year-old patient presented with progressive shortness of breath and tiredness. On admission, electrocardiography (ECG) demonstrated CHB with a ventricular rate of 38 bpm. Verapamil was withheld, and the patient was commenced on isoprenaline; a temporary pacing wire was not tolerated. During admission, he developed a right middle cerebral artery (MCA) infarct secondary to an M2 thrombus, resulting in expressive aphasia. No atrial fibrillation or left ventricular thrombus was identified, raising suspicion of septic embolism. Despite persistently negative blood cultures, echocardiography revealed a mobile structure within the aortic root near the non-coronary cusp with trivial regurgitation, and a mobile mass on the posterior mitral leaflet, though difficult to define due to calcification. According to the Modified Duke’s Criteria, the case met one major criterion (positive echocardiographic findings) and three minor criteria (predisposing heart disease, vascular event, clinical features), consistent with likely infective endocarditis. The patient was treated empirically with IV amoxicillin, flucloxacillin and gentamicin. From AMS perspective, broad-spectrum therapy was regularly reviewed by the multidisciplinary team, with toxicity monitoring and consideration of diagnostic refinement to enable targeted treatment.

**Conclusions:**

This case demonstrates the diagnostic complexity of CNIE. Although blood cultures were persistently negative, Duke’s Criteria and multimodal imaging supported the diagnosis. From an AMS perspective, key recommendations include:

- Optimizing microbiological sampling and using molecular diagnostics before initiating broad-spectrum therapy.
- Applying guideline-directed empirical regimens with early stewardship review and de-escalation where possible.
- Close collaboration between microbiology, cardiology, neurology and infectious diseases to tailor therapy to optimize antibiotic use and decrease AMR.
- Careful monitoring of nephrotoxic combinations (e.g. aminoglycosides) and considering oral or less toxic alternatives in frail patients.
